# Does feedback of medication execution using MEMS caps aid adherence to HAART?: the MEMRI study (MEMS as Realistic Intervention)

**DOI:** 10.1186/1758-2652-13-S4-P120

**Published:** 2010-11-08

**Authors:** S Davies, S Asghar, V Cooper, A Lange, C Robertson, B Vrijens, DJ White

**Affiliations:** 1 Birmingham Heartlands Hospital, Birmingham, UK; 2 The School of Pharmacy, University of London, London, UK; 3 Aardex Group Ltd., Sion, Switzerland

## Purpose of the study

Medication adherence is crucial for successful Highly Active Antiretroviral Treatment (HAART). Adherence may be divided into execution (how dosing history corresponds to the prescribed drug dosing regimen) and persistence (the time from the first to last taken dose). Medical Electronic Monitoring System (MEMS) monitors record bottle opening events providing a graphical printout of adherence. This can be used to provide positive feedback and correct any perceptual inaccuracies as to adherence. MEMRI assesses the use of such feedback as an intervention to support adherence. The primary endpoint is based on execution.

## Methods

265 patients were recruited. 180 were suitable for randomisation. All subjects were attending for HIV outpatients at Birmingham Heartlands Hospital. MEMS cap data was available for analysis for 145 of these. 78 (Group A) were given regular feedback using graphical readouts by clinical staff predominantly pharmacists. 67 (Group B) were blinded to feedback and no graphical output was available. MEMS 6 monitors (LCD display) were used on the most frequently dosed component of the HAART regimen. MEMS were downloaded at each clinic visit. At time of this interim analysis 12 months of follow-up had been completed for all subjects.

## Summary of results

123 patients took qid regimens, 14 took bid regimens, and 8 took multiple regimens during monitoring each divided evenly between Group A and Group B. Medication execution was high in both groups (>90%) for those patients who continued using the MEMS cap. Feedback (Group A vs. B) was not associated with a significant difference in execution but execution improved over time. There was a larger drop-out rate in Group B vs. Group A (22 vs. 13 patients) although this was not statistically significant. Execution was significantly worse at weekends (p=0.0001).

## Conclusions

A preliminary analysis of the MEMRI study primary endpoint is presented. On limited follow-up at 12 months MEMS feedback showed no effect on medication adherence but this was only on patients with high initial adherence execution. This large adherence study includes a wide range of patients and may be able to extrapolate to other groups. Data based further follow-up will be presented and when complete the study will include analysis of other factors such as perceived needs and concern, self-efficacy and conscientiousness.

